# Cost-minimisation analysis of a treat-and-extend regimen with anti-VEGFs in patients with neovascular age-related macular degeneration

**DOI:** 10.1007/s00417-021-05359-x

**Published:** 2021-10-13

**Authors:** S. W. Quist, L. A. de Jong, F. van Asten, P. Knoester, M. J. Postma, R. D. Freriks

**Affiliations:** 1 Department of Health Sciences, University of Groningen, University Medical Center Groningen, Hanzeplein 1, Groningen, The Netherlands; 2 Asc Academics, Professor Enno Dirk Wiersmastraat 5, Groningen, The Netherlands; 3 Department of Ophthalmology, Radboud University Medical Center, Philips Van Leydenlaan 15, Nijmegen, The Netherlands; 4 Department of Pharmacy, Alrijne Hospital, Simon Smitweg 1, Leiderdorp, The Netherlands; 5 Department of Economics, Econometrics & Finance, University of Groningen, Faculty of Economics & Business, Nettelbosje 2, Groningen, The Netherlands

**Keywords:** Anti-VEGFs, Treat-and-extend regimen, Age-related macular degeneration, Cost minimisation

## Abstract

**Purpose:**

Although intraocular anti-vascular endothelial growth factors (anti-VEGFs) are effective as treatment of neovascular age-related macular degeneration (nAMD), the (economic) burden on the healthcare system is considerable. A treat-and-extend (T&E) regimen is associated with a lower number of injections without compromising the effectiveness and can therefore help optimise nAMD treatment. This study investigates the per-patient costs associated with nAMD treatment, when using aflibercept, bevacizumab, or ranibizumab with a T&E regimen.

**Methods:**

In this cost-minimisation model, the per-patient costs in the Netherlands were modelled using a healthcare payers’ perspective over a 3-year time horizon with the assumption that efficacy of treatments is similar. Additionally, the break-even price of the different anti-VEGFs was calculated relative to the cheapest option and injection frequency.

**Results:**

The injection frequency varied from 14.2 for aflibercept to 27.4 for bevacizumab in 3 years. Nonetheless, bevacizumab remains the cheapest treatment option (€14,215), followed by aflibercept (€18,202) and ranibizumab (€31,048). The medication covers the majority of the per-patient costs for aflibercept and ranibizumab, while administration covers the majority of the per-patient costs for bevacizumab. The break-even prices of aflibercept and ranibizumab are respectively €507 and €60.58 per injection. Brolucizumab was included in the scenario analysis and was more expensive than aflibercept (€20,446). Brolucizumab should reduce to 13.8 injections over 3 years to be as costly as aflibercept.

**Conclusion:**

Bevacizumab is the cheapest anti-VEGF treatment. The list prices of all anti-VEGFs should reduce to be as costly as bevacizumab. Aflibercept is the second-choice treatment and so far brolucizumab is not.

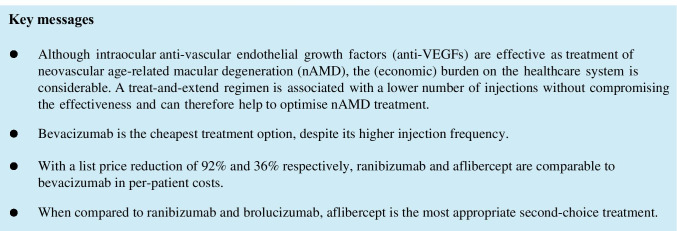



## Introduction

Age-related macular degeneration (AMD) is globally the main cause of severe vision loss [1]*.* In the period 2011–2018, the yearly prevalence of AMD in the Netherlands has increased by approximately 70% to 51,400 men and 81,600 women. It is expected that the prevalence will keep increasing as the aging of the population continues [2, 3].

AMD is characterised by the presence of drusen in the early stages but may progress into geographic atrophy or the neovascular form of AMD associated with severe vision loss (Van Leeuwen et al. 2003). Although only 10–15% of all AMD patients develop neovascular AMD (nAMD), it causes 90% of all AMD-related severe vision loss [4]. Due to its severity, the economic burden for the healthcare system caused by nAMD treatment is substantial. In the Netherlands, the hospital expenditures for AMD amounted to a total of €156 million in 2016 [5]. Moreover, anti-VEGF treatment often comes with a relatively high number of injections and monitoring visits, which causes a considerable burden for the patient and increasing waiting lists [6].

Intravitreal injections of anti-vascular endothelial growth factors (anti-VEGFs) are used as first-line treatment to prevent visual loss related to nAMD [7]. Currently, three anti-VEGFs are registered at the European Medicines Agency (EMA) for the treatment of nAMD: aflibercept, ranibizumab, and brolucizumab [8–10]. Bevacizumab is an anti-VEGF with an equal clinical effectiveness that is not officially registered for treatment of nAMD. However, in several countries, including the Netherlands, bevacizumab is since 2005 used as an off-label treatment for purpose of cost containment [7, 11–13]. In the Netherlands, bevacizumab is recommended as a first-choice treatment while the drug is still under review by the European Medicines Agency as treatment for nAMD [7, 11]. Clinical trials have shown no significant difference in effectiveness and safety between aflibercept, ranibizumab, and bevacizumab [14–18]. A fourth anti-VEGF, brolucizumab, was recently registered at the EMA, which has shown to be equally effective to the other anti-VEGFs. However, the anti-VEGF suspectedly leads to a higher risk of intraocular inflammations and retinal vasculitis [19]. Therefore, aflibercept and ranibizumab are currently second- and third-choice treatments and brolucizumab is a fourth-choice treatment according to the Dutch guideline for the treatment of AMD [20].

Multiple regimens for anti-VEGF treatment are currently being used for the treatment of nAMD. Earlier clinical trials focused on periodic regimens: for bevacizumab and ranibizumab a monthly regimen, and for aflibercept a bimonthly regimen. Brolucizumab was introduced with the periodic 12 weekly regimen, which was adjusted to an 8 weekly regimen in case of disease activity (Q12W/Q8W regimen) [18, 21, 22]. To reduce the number of injections during the anti-VEGF treatment, the flexible treat-and-extend (T&E) regimen was introduced. Trials with aflibercept, bevacizumab, and ranibizumab have shown that with a T&E regimen equal effectiveness and safety can be achieved with fewer injections [14, 15, 23–26]. T&E is a form of personalised medicine as the treatment and monitoring intervals can be extended (or shortened) based on the patient’s disease progression [14, 15]. The extension of the treatment interval depends on the patient and the anti-VEGF used. Although the medication costs per bevacizumab injection are much lower compared to those of the registered anti-VEGF injections, the number of injections needed to maintain optimal visual acuity outcomes with a T&E regimen seems to be lower for other anti-VEGFs [13, 14, 23, 27].

Optimisation of the T&E regimen could reduce pressure on the healthcare system and might be necessary as the number of people with ophthalmic disorders is expected to increase by 52% by 2040 [2].

To optimise the T&E regimen, it is important to consider all direct healthcare costs when choosing between the anti-VEGFs, and not solely medication costs, as done in the NOG guidelines. This cost-minimisation study aims to compare the per-patient costs within a T&E regimen with the different anti-VEGFs in treatment-naïve patients with nAMD in the Netherlands. Our study focusses on treatment-naïve patients to minimize the influences of former treatments and to include a broad spectrum of patients. Additionally, the break-even price for the different anti-VEGFs relative to the anti-VEGF with the lowest costs is determined.

## Methods

### Model characteristics

A cost-minimisation model was developed in Microsoft Excel 2016 (Redmond, WA, USA) to model the per-patient costs of the different anti-VEGFs for the treatment of nAMD. The costs were determined for a one-eye treatment of a treatment-naïve nAMD patient in the Netherlands when using a T&E regimen. Cost-minimisation analyses assume that the effectiveness and safety of the treatments under evaluation are comparable and therefore that treatments have no clinical benefits over each other [28]. This is not the case for brolucizumab given the suspected higher risks for intraocular inflammations and retinal vasculitis. Aflibercept, bevacizumab, and ranibizumab are comparable in effectiveness and safety and are therefore included in this study [7, 20].

The course of disease of nAMD was modelled based on published literature. The model included costs for medication, administration, diagnosis, clinic visits, and adverse events. The break-even prices of the anti-VEGFs with the highest total costs were calculated based on the anti-VEGF with the lowest total costs per patient as reference. The assumptions that were made in the base case scenario are shown in Table 1 and are further elucidated below.

**Table 1:** Overview of the assumptions made in the base case scenario

Model characteristics	• A cost-minimisation model was used because of the comparative effectiveness and safety of the drugs and similar patient characteristics in the clinical trials.
	• A Dutch healthcare payer’s perspective was used because direct costs are relevant for the daily practice, as the choice between treatments is made by hospitals and other treatment centres.
	• The used time horizon was 3 years to conform to the Dutch guideline for budget-impact analyses in healthcare [29].
T&E regimen characteristics	• The model focused on a T&E regimen, consisting of a loading phase and a maintenance phase:
	Loading phase: three times a month injection
	Maintenance phase: Treatment intervals could be either extended (in case of absence of intra- or subretinal fluid) or shortened (in case of presence of fluid) by 2 weeks after every monitoring visit. The minimal interval is 4 weeks and the maximal interval is 12 weeks
	• The patient monitoring was based on the NOG guideline [7]:
	During the loading phase, one diagnostic monitoring visit takes place and during the maintenance phase, a monitoring visit takes place for every injection
	During the first monitoring visit, the patient is diagnosed with an OCT, fundus photography, and FA
	In every subsequent monitoring visit, the patient is monitored with an OCT
	Injections and monitoring visits take place simultaneously
	• The injection frequency per anti-VEGF treatment was based on a weighted average of injection frequencies as reported in the phase III and/or IV clinical trials
	• The numbers of injections in the second and third years were assumed to be equal because all included clinical trials have a maximal time span of 2 years. This assumption is supported by real-world data showing that the number of injections in the second and third years is similar [30, 31, 33, 34]
Adverse events	• Injection-related adverse events with relatively high costs and prevalence were included
	• The risk per injection for these adverse events was equal for the different anti-VEGFs
Costs	• The medication costs of aflibercept and ranibizumab were based on the official list prices [35, 36]
	• The medication costs of bevacizumab were based on the costs of self-preparation by hospital pharmacists and calculated based on a previous cost calculation [13]

*anti-VEGF*, Anti-vascular endothelial growth factor; *FA*, Fluorescence angiography; *NOG*, Dutch Ophthalmological Society; *OCT*, Optical coherence tomography; *T&E*, Treat-and-extend

### Time horizon and perspective

We used the Dutch healthcare payer’s perspective, which entails all the direct healthcare-related costs in the Netherlands induced by anti-VEGF treatment of treatment-naïve nAMD patients. Direct costs are relevant for daily practice, as the choice between treatments is made by hospitals and other treatment centres. The per-patient costs and break-even prices were calculated for 3 years according to the Dutch guideline for budget-impact models in healthcare [29].

### T&E regimen characteristics

NOG issued a guideline for the use of aflibercept, bevacizumab, and ranibizumab. Bevacizumab was chosen as the preferred treatment option because of the relatively low medication costs [7]. Aflibercept and ranibizumab are second- and third-choice treatments. Brolucizumab received market authorisation in February 2020 and is therefore not yet included in the NOG guideline [9]. However, the NOG recently published a statement about the proposed position of brolucizumab in the Dutch treatment guideline. The NOG recommended brolucizumab as a fourth-choice treatment because of the higher risk for intraocular inflammation and retinal vasculitis [20].

According to the T&E regimen in the NOG guideline, all patients should start with a loading dose of three times a month injection, followed by flexible treatment intervals which will be extended based on the treatment response, which is determined on the hand of (an increase of) intra- or subretinal fluid. Treatment intervals could be either extended (in case of absence of intra- or subretinal fluid) or shortened (in case of presence of fluid) by 2 weeks after every monitoring visit. The minimal interval is 4 weeks and the maximal interval is 12 weeks [7].

The injection frequency input data included in the model were based on phase III and/or IV, prospective, randomised trials. Trials should study one-eye anti-VEGF treatment with a T&E regimen in treatment-naïve nAMD patients. The primary endpoint of the trial should focus on the improvement in vision of patients. A weighted average of the injection frequency was used when multiple trials were found for the same anti-VEGF. A summary of the included regimens and the clinical trials is shown in Table 2. Detailed overviews of each included trial can be found in Appendix 1 (Tables 8 and 9). All performed clinical trials have a maximum interval period of 2 years. From real-world data, it becomes apparent that the number of injections in the second and third years is similar [30, 31, 33, 34]. It was therefore assumed that the number of injections in the second year is equal to the number of injections in the third year.

**Table 2:** Overview base case regimens for the different anti-VEGFs

Anti-VEGF	Description of the treatment regimen	Source
Aflibercept	A loading dose was used. After this, the minimal treatment interval was 8 weeks and the maximal treatment interval was 16 weeks. The treatment intervals were extended or shortened by 2-week periods	*ALTAIR* [23]
Bevacizumab	No loading dose was administrated. The minimal treatment interval was 4 weeks and the maximal treatment interval is 12 weeks. The treatment intervals were extended or shortened by 2-week periods	*LUCAS trial* [14, 39]
Ranibizumab	LUCAS trial: no loading dose was administered. Patients directly started with a T&E regimen TREX, TREND, and CANTREAT trials: a loading dose was used All trials: Minimal treatment interval in all trials was 4 weeks and maximal treatment interval was 12 weeks. The treatment intervals were extended or shortened by 2-week periods	*LUCAS, TREX, TREND, and CANTREAT* [14, 25, 26, 39–41]

*T&E*, treat-and-extend

During the loading phase, we assumed one monitoring visit. During the maintenance phase, one monitoring visit is assumed for every injection [7]. In year 1, two fewer monitoring visits were assumed than in the following years because of the single monitoring visit during the loading phase. The reported injection frequencies and correlating monitoring frequencies are shown in Table 3. Additionally, Appendix 1, Table 10, provides an overview of the number of injections before time corrections and calculation of the weighted average, as reported in each trial.

**Table 3:** Overview of the injection and monitoring frequency per year used in the model

	*Injection frequency*				*Monitoring frequency*				*Sources*
	Year 1	Year 2	Year 3	Total	Year 1	Year 2	Year 3	Total	
Aflibercept^**a**^	7.2	3.5	3.5	14.2	5.2	3.5	3.5	12.2	*ALTAIR* [23]
Bevacizumab	9.0	9.2	9.2	27.4	7.0	9.2	9.2	25.4	*LUCAS trials* [14, 39]
Ranibizumab	9.0	8.2	8.2	25.4	7.0	8.2	8.2	23.4	*LUCAS, TREX, TREND, and CANTREAT* [14, 25, 26, 39–41]

^a^The ALTAIR trial lasted 96 weeks. This time span was corrected to 24 months to align all results.

### Adverse events

The four most prevalent adverse events were included in the model by risk per injection. Since all the adverse events included in this model are solely injection-related, the risk per injection was equal for all three anti-VEGFs. Moreover, none of the clinical trials found a significant difference in injection-related adverse events [14, 15, 17, 18, 21, 22]. The included adverse events with corresponding risks are shown in Table 4.

**Table 4:** Risk per injection for adverse events

	Risk per injection	Source
Endophthalmitis	0.0004	[42]
Retinal detachment	0.0001	
Lens injury	0.0001	
Intraocular haemorrhage	0.0003	

### Costs

The per-patient costs were calculated by taking the sum of all the direct healthcare costs related to treatment of one nAMD patient. All costs have been adjusted for inflation to November 2020 [43]. All the implemented costs are summarised in Table 5.

**Table 5:** Overview of all the included costs for the different anti-VEGFs

	Dose (mg)	Costs per quantity	Cost year	Source
Medication and administration costs				
Aflibercept^a^	2.00	€789	2020	[36]
Bevacizumab	1.25	€17.15	2020, 2008	[13, 27, 46]
Ranibizumab^b^	0.50	€726	2020	[35]
Administration costs (all drugs)	-	€368	2012	[44]
Clinic visit costs				
Polyclinical visits	-	€89.97	2012	[44]
OCT (diagnosis and monitoring)	-	€44.71	2012	[44]
FA (diagnosis)	-	€84.39	2012	[44]
Fundus photography (diagnosis)	-	€44.11	2020	[45]
Adverse event-related costs				
Endophthalmitis	-	€ 3839	2012	[44]
Retinal detachment	-	€ 2496	2012	[44]
Lens injury	-	€ 1889	2012	[44]
Intraocular haemorrhage	-	€ 249	2012	[44]

*Abbreviations*: *anti-VEGFs*, anti-vascular endothelial growth factor; *FA*, fluorescence angiography; *OCT*, optical coherence tomography

^a^The Z-index prices were elevated by 9% because of the value added tax rate in the Netherlands [47]

^b^Declaration code: 39,917.

The medication costs of aflibercept and ranibizumab were based on the list prices [35, 36]. No official list prices of standardised bevacizumab intravitreal injections were available, since it is used off-label. The price of an in-hospital prepared injection of bevacizumab was calculated based on a previous publication [13]. An overview of these calculations is shown in Appendix 2 (Tables 11, 12, 13).

Some hospitals order fully prepared bevacizumab intravitreal injections by production pharmacies, which are more expensive compared to the preparation in the hospital itself. The effect of the use of this more expensive preparation was tested in the scenario analysis.

For every injection, administration costs were taken into account. Administration costs were based on a previously published economic evaluation of anti-VEGFs in the Netherlands and consisted of all the costs corresponding to the injection (except medication costs) [44]*.* The utilisation of the monitoring visits was based on the NOG guideline. The diagnosis consists of optical coherence tomography (OCT), fundus photography, and fluorescence angiography (FA). Subsequent monitoring visits include an OCT only [7]. The OCT and FA costs were both based on the study of Elshout et al. [44]. The fundus photography costs were based on the Dutch Healthcare Authority (NZA) [45]. The costs of adverse events were also based on the study of Elshout et al. [44].

### Break-even price

After the determination of the per-patient cost, the break-even price of every anti-VEGF relative to the anti-VEGF with the lowest total costs was calculated. This is the drug price of the relatively more expensive anti-VEGFs to reach equal per-patient total costs as for the cheapest anti-VEGF when using a T&E regimen. The break-even price is calculated by subtracting the sum of the administration, clinic visit costs, and adverse event-related from the total per-patient costs of the cheapest anti-VEGF. This is divided by the total injection frequency, resulting in the break-even price per injection. Additionally, the difference between the break-even price and the list price was presented as the percentage reduction (discount). To determine the effect of deviating injection frequencies, also the break-even price relative to the injection frequency was calculated.

### Univariate sensitivity and scenario analyses

A univariate sensitivity analysis was performed to assess the influence of the different model input parameters on the total costs per patient per anti-VEGF. The parameters were variated with an interval of + / − 25% [48]. Additionally, two scenario analyses were performed: (1) bevacizumab costs based on the purchase price of injections prepared by production pharmacies and (2) brolucizumab was included as treatment option (Table 6). As no T&E regimen data was available, brolucizumab was included using a Q12W/Q8W regimen. In this regimen, the patient receives an injection every 12 or 8 weeks after the loading dose, depending on the disease progression [22]. Besides calculating brolucizumab’s break-even price, its per-patient costs relative to the injection frequency were plotted against the anti-VEGF with the second lowest costs.

**Table 6:** Overview of the different performed scenario analyses

	Description
Scenario 1	**Bevacizumab medication costs based on fully prepared injections** Instead of self-preparation at hospital pharmacies, it was assumed that bevacizumab injections were ordered at production pharmacies. Therefore, the medication price in this scenario was adjusted to the (higher) purchase price for these bevacizumab injections [27]
Scenario 2	**The per-patient costs for brolucizumab were calculated together with the maximal allowed adverse event costs** Brolucizumab was not considered as a treatment option in the base case scenario since it is a fourth-choice treatment in the Netherlands due to the increased chance for retinal vasculitis. Moreover, cost-minimisation analyses assume that the effectiveness and safety of the treatments under evaluation are comparable In this scenario, the injection frequency for brolucizumab was based on the HAWK and HARRIER trial, which uses a Q12W/Q8W-regimen. After the loading dose, the patient receives an injection every 12 weeks which could be adjusted to an 8 weekly regimen in case of disease activity [22, 49]. The trial characteristics can be found in Appendix 1 (Tables 8 and 9). The injection frequency can be found in Appendix 3 (Table 14). In total, 15.4 injections over 3 years were included. The medication costs of brolucizumab are €825 and can be found in Appendix 3 (Table 15). All other costs included are equivalent to the costs for the other anti-VEGFs. After calculating the per-patient costs, it is determined how much the adverse event costs can be elevated to be similar in costs to the other anti-VEGFs

*anti-VEGF*, anti-vascular endothelial growth factor; *Q12W/Q8W*, every 12 weeks/every 8 weeks

## Results

### Base case scenario

#### Per-patient costs

**Fig. 1:**
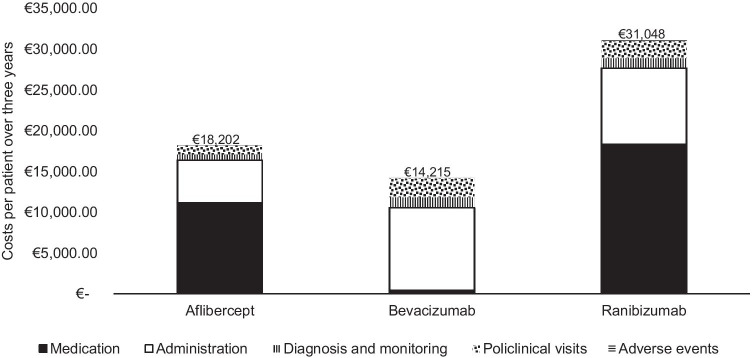
Per-patient costs per anti-VEGF over a 3-year time horizon in the base case analysis. Abbreviations: anti-VEGF, anti-vascular endothelial growth factor

Solely the per-patient costs of the anti-VEGFs are compared as clinical trials show that despite their different treatment intervals, they pursue an equal clinical effect [23–26, 30, 39]. Figure 1 and Table 7 show the per-patient costs when using the different anti-VEGFs for nAMD over a 3-year time horizon. Bevacizumab use is associated with the lowest total per-patient costs (€14,215). After bevacizumab, aflibercept has the lowest total per-patient costs of €18,202, followed by ranibizumab with €31,048 per patient. The medication costs are the majority of the total per-patient costs for aflibercept or ranibizumab (61.4% and 59.2%, respectively). Whereas for bevacizumab, the administration costs form most of the total per-patient costs (71.0%). The medication costs account for 3.3% of the total per-patient costs for bevacizumab. The lowest share of costs for all three anti-VEGFs is the adverse event-related cost (varying from 0.3 to 0.7%).

**Table 7:** The per-patient costs over a 3-year time horizon and the break-even price per injection in the base case and scenario analyses

Costs per patient	Medication	Administration	Diagnosis and monitoring	Clinic visits	Adverse events	Total	Break-even price vs bevacizumab
Base case							
Bevacizumab	€470	€10,093	€1264	€2285	€102	**€14,215**	**Reference**
Aflibercept	€11,168	€5215	€672	€1,094	€52.66	**€18,202**	**€507 (− 36%)**
Ranibizumab	€18,365	€9321	€1170	€2097	€94.12	**€31,048**	**€60.58 (− 92%)**
Scenario 1: Bevacizumab medication costs based on fully prepared injections							
Bevacizumab	€972	€10,093	€1264	€2285	€102	**€14,717**	**Reference**
Aflibercept	€11,168	€5215	€672	€1094	€52.66	**€18,202**	**€542.72 (− 36%)**
Ranibizumab	€18,365	€9321	€1170	€2097	€94.12	**€31,048**	**€80.42 (− 89%)**
Scenario 2: The per-patient costs for brolucizumab were calculated together with the maximal allowed adverse event costs							
Bevacizumab	€470	€10,093	€1264	€2285	€102	**€14,215**	**Reference**
Brolucizumab	€12,756	€5693	€730	€1210	€57.48	**€20,446**	**€422 (− 49%)**
Aflibercept	€11,168	€5215	€672	€1094	€52.66	**€18,202**	**€507 (− 36%)**
Ranibizumab	€18,365	€9321	€1170	€2097	€94.12	**€31,048**	**€60.58 (− 92%)**

#### Break-even price

Treatment with bevacizumab results in the lowest total per-patient costs and the break-even price is therefore calculated relative to bevacizumab. The break-even price is the drug price per injection that leads to equivalent per-patient costs as bevacizumab. The results are shown in Table 7. Ranibizumab has the lowest break-even price (€60.58) and should decrease by 92% in medication price to reach equal costs. The break-even price of aflibercept is €507 and should decrease by 36% to reach equal costs.

**Fig. 2:**
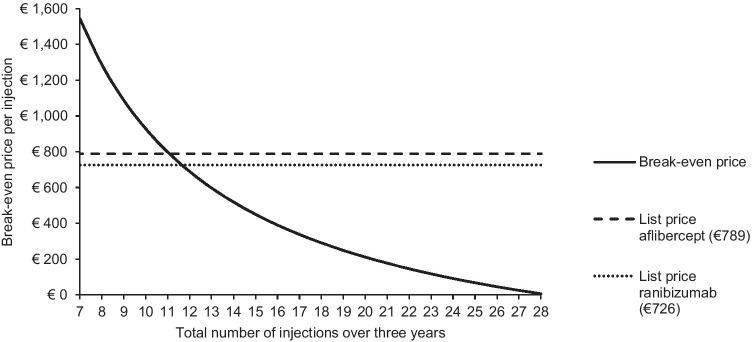
Break-even price per injection relative to the injection frequency over 3 years

Figure 2 displays the break-even price relative to the injection frequency. A total injection frequency above 28 over a 3-year time horizon outreaches the injection frequency of bevacizumab and brings the break-even price below 0. With the current list prices of aflibercept and ranibizumab, respectively, 11.1 and 11.7 injections over 3 years are needed to achieve equal per-patient costs to bevacizumab.

### Univariate sensitivity analysis

The outcomes of the univariate sensitivity analysis are presented in tornado diagrams (Appendix 4). For all three anti-VEGFs, it is visible that the injection frequency has the greatest influence on the per-patient costs. Therefore, the injection frequency strongly affects the break-even prices. Varying the injection frequency of aflibercept by 25% (varying from 10.6 to 17.7 in 3 years) leads to per-patient costs corresponding with a break-even price varying from €845 to €304. Varying the injection frequency of ranibizumab by 25% (varying from 19.1 to 31.8 in 3 years) leads to per-patient costs corresponding with a break-even price variating from €245 and − €55.42. Varying the injection frequency of bevacizumab by 25% also affects the break-even price of aflibercept and ranibizumab. For aflibercept, the break-even price variates between €254 and €761 and for ranibizumab the break-even price variates between − €81.26 and €202.

For bevacizumab, the intravitreal injection costs have the second biggest influence while for the other anti-VEGFs the medication costs have the biggest influence. Other important costs are the administration costs, the costs for clinical visits, and OCT costs.

### Scenario analyses

#### Scenario 1

Instead of self-preparation, some hospitals order fully prepared bevacizumab injections. Therefore, the scenario of ordering fully prepared bevacizumab injection was performed. As is visible in Table 7, the per-patient costs of bevacizumab in scenario 1 are slightly higher compared to the base case scenario. However, the total costs per patient are still lower than those for all the other anti-VEGFs.

#### Scenario 2

**Fig. 3:**
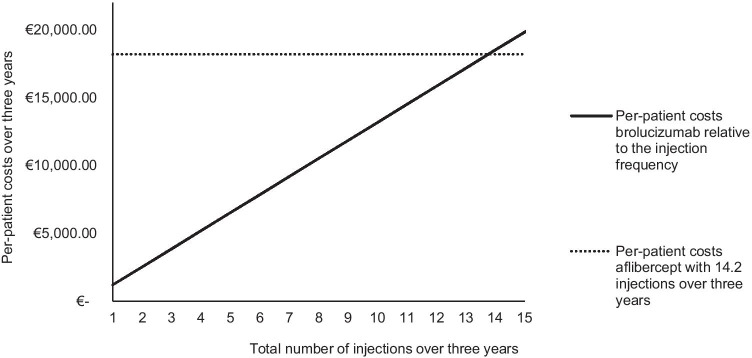
The per-patient costs of brolucizumab relative to the injection frequency plotted against the total per-patient costs of aflibercept with 14.2 injections

Scenario 2 assessed the per-patient costs of treatment with brolucizumab (€20,446). The per-patient costs and break-even price of brolucizumab are higher than those of aflibercept because of the slightly higher injection frequency in the Q12W/Q8W regimen (Table 7). With higher adverse event costs, brolucizumab will be even more expensive than aflibercept. In Fig. 3, the per-patient costs of brolucizumab relative to the injection frequency are plotted against the per-patient costs of aflibercept with the injection frequency of the ALTAIR trial. With an injection frequency of 13.8, the per-patient costs of brolucizumab and aflibercept are equal.

## Discussion

Our study shows that despite its high injection frequency, bevacizumab is the cheapest treatment option for treatment-naïve nAMD patients, with per-patient costs of €14,215 per patient over a 3-year time horizon. This was mainly caused by the relatively low medication costs compared to those of aflibercept and ranibizumab. Bevacizumab is the only anti-VEGF with higher administration costs than medication costs. Aflibercept and ranibizumab lead to total per-patient costs of €18,202 and €31,048, respectively. The break-even prices of ranibizumab and aflibercept were respectively €60.58 and €507, which are 92% and 36% below their current list price. Aflibercept is cheaper than ranibizumab due to its relatively low injection frequency.

Based on the list prices, bevacizumab seems the most economically favourable option. Choosing either aflibercept or ranibizumab will therefore lead to higher healthcare-related costs. However, by calculating the break-even prices, we aimed to provide insight into the necessary discounts to reach cost equality for the anti-VEGFs. Besides the difference in costs, the non-economical effect of a high injection frequency is also an important factor to consider when choosing between the anti-VEGFs. Intravitreal injections lead to anxiety in patients and the clinical visits are a burden on the patient as well [6].

To optimise nAMD treatment for the patient and the healthcare system, this study focuses on a T&E regimen. In a flexible T&E regimen, fewer injections are needed than in fixed regimens to guarantee similar effectiveness. The effectiveness of a T&E regimen is shown to be higher than that of other flexible regimens [50, 51]. A recent discrete-choice experience amongst Japanese patients showed that in most cases, patients prefer a T&E regimen over other regimens because of the higher chance for improvement in the visual acuity of a T&E regimen [52]. Moreover, using a T&E regimen with an anti-VEGF with a low injection frequency also helps reduce the pressure on the healthcare system. Ophthalmology is currently the hospital specialism with the longest waiting lists in the Netherlands and the waiting lists are expected to continue to grow due to the aging population [3, 53, 54]. Also, the current COVID-19 crisis is predicted to further reduce capacity. During the first peak, fewer people went to see a general practitioner for issues unrelated to COVID-19 and treatment of several eye-related illnesses was postponed. Both trends led to increasing pressure on the hospitals later [55, 56]. Moreover, elderly patients might be scared to visit the hospital during the global pandemic because of the risk of being exposed to the virus. By focusing on cost-minimisation of the T&E regimen, we hope to stimulate the use of this regimen.

In the scenario analysis, it was shown that brolucizumab was, with 15.4 injections over 3 years, more expensive than aflibercept but cheaper than ranibizumab. No trials with a T&E regimen were available for brolucizumab; and therefore, these outcomes are based on the Q12W/Q8W regimen. Our results showed that in a T&E regimen, brolucizumab would be cheaper than aflibercept with fewer than 13.8 injections in 3 years. However, brolucizumab is clinically the fourth choice of treatment because of the suspected higher chance of intraocular inflammation. These adverse events were not included in our analysis due to a lack of clinical data. At the time of this study, the TALON trial was still ongoing [57]. This trial focuses on the T&E regimen for brolucizumab and aflibercept. For future studies, it would be interesting to include this trial. Hopefully, the TALON study will bring more knowledge about the injection frequency of brolucizumab in a T&E regimen, and the increased risk of brolucizumab for inflammatory eye-reactions including its associated costs, and therefore the position of brolucizumab relative to the other anti-VEGFs.

Using the slightly more costly pre-filled bevacizumab injections in the scenario analysis still led to lower per-patient costs for bevacizumab compared to the other anti-VEGFs. The preparation method of bevacizumab seems to have little impact on the per-patient cost. The fully prepared bevacizumab injections were only €502 more expensive than the self-prepared injections over 3 years.

Several pharmacoeconomic studies compare the cost-effectiveness between (some of) the different anti-VEGFs. However, this study is the only known pharmacoeconomic study that focuses on a T&E regimen and cost-minimisation. In most studies, bevacizumab has shown to be more cost-effective than ranibizumab or aflibercept because of its lower medication price and equivalent effectiveness [58–60]. This is in line with the results of our study. Aflibercept is often perceived as a more cost-effective option than ranibizumab because of the lower injection frequency shown in clinical trials [58, 60–63]. This is also in line with the results of our study, although it should be noted that it is not possible to directly compare the cost-effectiveness analyses to our study because we focus solely on costs. Another difference is that the other studies based their injection regimens on one single clinical trial or assumed a non-flexible regimen, whereas we focus on the T&E regimen.

As shown in the univariate scenario analysis, injection frequencies are the main driver for the total per-patient costs. The injection frequencies used in the model were based on previously published phase III and IV clinical studies. However, the use of different sources has some implications. For example, the included trials used different patient populations, which might reduce the comparability of their results. The ALTAIR trial is a Japanese trial that includes Asian patients, whereas all other trials were conducted in Europe or North America. nAMD characteristics for Asian patients differ from those for white patients. A higher proportion of the polypoidal choroidal vasculopathy subtype and a lower prevalence of retinal angiomatous proliferation are seen in Asian patients than in white patients. It is unclear whether ethnicity impacts treatment outcomes, and thus if the ALTAIR trial outcomes are comparable with the other studies. The VIEW study compared aflibercept and ranibizumab treatments for both white and Asian patients. In a sub-analysis, there were almost no significant differences found between the patient groups. This study used a fixed regimen in the first year and a flexible Pro Re Nata regimen in the second year. The number of injections was similar for both patient groups [64]. In a Japanese real-world study, the injection frequency for polypoidal vascular AMD patients is compared to typical AMD patients. In this bi-monthly and T&E regimen, the injection frequency over 3 years was nearly equal for both patient groups (17.4 ± 3.7 for typical AMD and 17.1 ± 2.6 for polypoidal AMD) [34].

All included studies analysed the effects of a T&E regimen but there were some differences within the regimens. Some of the regimens followed in the clinical trials deviate from the NOG guidelines (e.g. the LUCAS and ALTAIR study) [14, 15, 23]. For example, the ALTAIR trial uses a minimal treatment interval of 8 weeks and a maximal treatment interval of 16 weeks, which may have led to an underestimation of the injection frequency. The differences between studies lead to insecurities in our model. We calculated the break-even price with the best available evidence, but our injection frequency might deviate from practice. Therefore, our study shows how the break-even price is affected by different injection frequencies.

Although the RIVAL trial focused on a T&E regimen for aflibercept and ranibizumab, this trial is not included in our study. The RIVAL used geographic atrophy as primary endpoint and visual improvement and number of injections as secondary endpoints. The trial found more injections for aflibercept in a T&E regimen than in a bimonthly regimen, while a bimonthly regimen is the fixed regimen used for aflibercept [10]. Moreover, the RIVAL trial shortened the treatment interval to 4 weeks if more than one sign of disease activity was found. The primary endpoint and the deviant treatment regimen reduce the external validity.

Our study does not include real-world studies because not for all anti-VEGFs 2-year T&E regimen real-word data was available. The injection frequency differs strongly between studies but the weighted average showed a similar injection frequency for aflibercept in the first year and a higher injection frequency for aflibercept in the second year than the ALTAIR trial (respectively 7.3 and 5.0 in real-world data) [23, 30–32, 37, 38]. For ranibizumab, only 1-year data was available but the weighted average of the real-world data showed a comparable number of injections as included in our study (8.7 injections) [50, 65–67]. Unfortunately, there was only one known real-world study for bevacizumab with a T&E regimen. This study found an injection frequency of 6.1 in the first year but the patients switched to ranibizumab in the second year [68]. A recent real-world study in the Netherlands looked into the use of anti-VEGF as a treatment for nAMD. All patients started with bevacizumab and some of the patients switched to aflibercept and ranibizumab. The total injection frequency was 26 over 3 years, similar to the total bevacizumab and ranibizumab frequency in this study but higher than the total aflibercept frequency. Unfortunately, it is not possible to compare their injection frequencies with this study because the study used an average of all treatment regimens and all three anti-VEGFs [69].

Although clinical trials suggest some (serious) non-injection-related adverse events, only the injection-related adverse events are considered in the model. This assumption was made because of the relatively small influence of adverse events costs, as shown in the univariate sensitivity analysis. Moreover, the risks for non-injection-related adverse events are considerably small and more importantly equivalent for aflibercept, bevacizumab, and ranibizumab [14, 15, 17, 18, 21, 22, 42]. Therefore, it is expected that this affects the results insignificantly.

The major strength of this study is that it considers all available anti-VEGFs. Moreover, the study gives a clear overview of the differences in the (distribution of) costs and break-even prices. The focus on a T&E regimen is also a strength since this is likely to be the leading regimen in the future of nAMD treatment in the Netherlands. This study gives a realistic insight on the difference between the actual per-patient costs when using the different treatments, and thus contributes to the needed knowledge for making a more considerate choice between the use of the different anti-VEGFs.

In conclusion, bevacizumab is the cheapest treatment option based on list prices. When considering the difference in injection frequency with a T&E regimen, the costs of treatment with aflibercept and ranibizumab will be cost neutral compared to bevacizumab at prices per injection of €507 and €61, respectively. Brolucizumab should reduce from 15.4 injections in a Q12W/Q8W regimen to 13.8 injections over 3 years in a T&E regimen in order to be as costly as aflibercept. Therefore, aflibercept is the second-choice treatment and so far there is no reason to consider brolucizumab as the second choice.

## Supplementary Information

ESM 1 (DOC 70.0 KB)

ESM 2 (DOC 45.0 KB)

ESM 3 (DOC 32.0 KB)

ESM 4 (DOCX 28.0 KB) 
