# Supplementary material for: Cost-minimisation analysis of a treat-and-extend regimen with anti-VEGFs in patients with neovascular age-related macular degeneration
Source: Graefes Arch Clin Exp Ophthalmol. 2021 Oct 13;260(4):1083–95. doi: 10.1007/s00417-021-05359-x (PMC8511619; doi:10.1007/s00417-021-05359-x)
Supplement: Supplementary file 1 — (DOC 70.0 KB) [file 417_2021_5359_MOESM1_ESM.doc]

## Appendix 1

**Table A1 Overview of the phases, set-ups, duration, included drugs and regimes, and primary endpoints of the included clinical trials.**

| **Clinical trial** | **Phase** | **Set-up** | **Duration** | **Drug(s) and regime(s)** | **Primary end point(s)** | **Source** |
| --- | --- | --- | --- | --- | --- | --- |
| **TREX** | Phase IIIb | Randomized, controlled, multi-center study | 24 months | Ranibizumab (monthly and T&E^a^) | Mean BCVA change from baseline | [23, 40] |
| **LUCAS** | Phase IIIb | Randomized, double masked, multi-center study | 24 months | Ranibizumab and bevacizumab (T&E, without loading dose) | Mean BCVA change as measured on the ETDRS chart. | [13] |
| **TREND** | Phase IIIb | Randomized, assessor-masked, multi-center study | 12 months | Ranibizumab: T&E regime and monthly regime | Demonstrate the noninferiority of ranibizumab in a T&E regime relative to a monthly regime. | [24] |
| **CANTREAT** | Phase IV | Randomized, open-label, multi-center, noninventory, post authorization study | 24 months | Ranibizumab (monthly)  Ranibizumab (T&E) | The effectiveness of 2 treatment regimens by assessing the mean change in BCVA (ETDRS letters) from baseline to month 24 | [25, 41] |
| **ALTAIR** | Phase IV | Randomized, open-label, multi-center study | 96 weeks | Aflibercept: T&E regimes with 2 weeks lengthening/shortening of the treatment interval  T&E regime with 4 weeks lengthening/shortening of the treatment interval | Change in BCVA letters from baseline | [22] |
| **HAWK/HARRIER (scenario 2)** | Phase III | Randomized, double-blinded, multi-center study | 96 weeks | Brolucizumab (q12w/q8w)  Aflibercept (fixed q8w) | BCVA from baseline, proportion of patients on q12w, retinal fluid changes and safety | [21, 49] |

**Abbreviations**: BCVA, best corrected visual acuity; ETDRS, Early Treatment Diabetic Retinopathy Study ; T&E, treat and extend
^a^ Unless indicated otherwise, every T&E regime is started with a loading dose and the treatment intervals hereafter are shortened/prolonged with two weeks.

**Table A2 Overview of the inclusion criteria of the patients and criteria for reinjections used in the different clinical trials**

| **Trials** | **Inclusion criteria patients** | **Criteria for reinjection** | **Source** |
| --- | --- | --- | --- |
| **TREX** | Untreated nAMD patients with choroidal neovascularisation. Subretinal haemorrhage and fibrosis comprising less than 50% of the total lesion.  BCVA: 78-18 ETDRS.  *(all patients were above age of 50 but is not noted as inclusion criteria)* | Patients were classified with wet or dry AMD by resolution of intraretinal and subretinal fluid and haemorrhage. In case of dry AMD the interval was prolonged with 2 weeks.  In case of wet AMD the interval shortened with 2 weeks, the interval hereafter is extended by 1 week. When a wet macula was again detected the interval was shortened with 1 week. When an eye showed a wet macula in 3 subsequent visits, the interval was shortened and kept at that interval 3 times in a row.  Maximal treatment interval: 12 weeks  Minimal treatment interval: 4 weeks   - **The interval after recurrent disease is only prolonged with 1 week** | [23, 40] |
| **LUCAS** | Untreated one eye nAMD patients above the age of 50 (choroidal leakage involving the center of the macula and intraretinal and subretinal fluid)  BCVA: 20/25 – 20/320 (80-between 30 and 20 letters) | Patients examined every 4 weeks until no signs of active AMD were found (determined by OCT and biomiscropic fundus examinations). When there were no signs the treatment interval was prolonged by 2 weeks. By recurrent disease the interval was shortened with 2 weeks,  Recurrent disease: any fluid on OCT, new or persistent haemorrhage and dye leakage, or increased lesion size on FA. Decreased BCVA was not defined as recurrence.  Maximal treatment interval: 12 weeks  Minimal treatment interval: 4 weeks.   - **The maximal interval after recurrent disease this is 2 weeks shorter than the interval leading to recurrent disease**. | [13] |
| **TREND** | Untreated patients above the age of 50 with visual impairment resulting from active CNV secondary to AMD confirmed by presence of active leakage of CNV. Total area of fibrosis less than 50%.  BCVA: 78-23 ETDRS | Patients were treated with monthly intervals until disease activity was resolved, as assessed by OCT and the investigator’s judgement. The interval could be prolonged with 2 weeks after every control. When disease activity was present, the interval could be shortened with 2 weeks. The possibility to extend the intervals between treatments was limited to 2 attempts. If disease activity recurred, the visit schedule was shortened by 2 weeks and fixed on this interval up to the end of the study.  Maximal period: 12 weeks  Minimal period: 4 weeks   - **If disease activity was present along with visual impairment, the treatment interval was allowed to shorten by 4 weeks instead of 2 weeks based on the investigator’s judgment** | [24] |
| **CANTREAT** | Untreated patients above the age of 50 with a diagnosis of treatment-naive choroidal neovascularization secondary to AMD in the study eye  BCVA: 78-19 letters | Treatment interval was shortened when:  The presence of any fluid, vision loss of more than 5 ETDRS letters, presence of new haemorrhage or progression of choroidal neovascularization, or a combination thereof.  After shortening interval: patient had to show no disease activity 2 times in a row to extend interval. If the patient showed disease activity again the interval was not prolonged for the whole study period.  **Maximal treatment period: 12 weeks**  **Minimal treatment period: 4 weeks** | [25, 41] |
| **ALTAIR** | Untreated patients above the age of 50 with exudative changes due to active CNV lesions secondary to AMD, including juxtafoveal lesions that affected the fovea.  BCVA: 73-24 ETDRS | When any of the following criteria are met for the study eye, the treatment interval was shortened:  -New or persistent fluid with unchanged or increased fluid volume from measurement at the previous treatment visit as indicated by OCT  -Loss of ≥ 5 ETDRS letters from the previous visit in conjunction with recurrent fluid on OCT  -An increase in CRT of ≥ 100 μm at the central 1 mm compared with the lowest previous value measured by OCT  - New-onset neovascularization as determined at the investigator’s discretion based on review of fundus examination and multi-imaging assessment if deemed necessary  - New macular haemorrhage  - New fluid or persistent intra- or subretinal fluid with unchanged or increased fluid volume from the previous visit as indicated by total OCT scan area (all volumetric fluid assessments were derived from multiple cross-sectional images and extracted from the OCT report)  If none of the criteria for shortening were met and residual fluid had decreased from the previous visit, then the treatment interval was maintained without change, even with persistent fluid  If none of the criteria for shortening were met and there was no fluid on OCT, then the interval was extended  The regime where the treatment interval was prolonged/shortened with two weeks was used in the model.  **Maximal treatment period: 16 weeks**  **Minimal treatment period: 8 weeks**  ***More than half of patients had a treatment interval longer than 12 weeks.*** | [22] |
| **HAWK/HARRIER (scenario 2)** | Untreated patients above the age of 50 with choroidal neovascularization lesions secondary to AMD affecting the central subfield. Also, choroidal neovascularization lesions affecting >50% of total lesion area, IRF/SRF affecting central subfield.  BCVA: 78-23 ETDRS | When disease activity was identified, patients switch permanently to 8-weekly treatment.  The disease assessment criteria were  At week 16: decrease of ≥5 letters in comparison with baseline  decrease of ≥3 letters and CTS increase ≥75 µm compared with week 12  decrease of ≥5 letters due to nAMD activity compared to week 12  new or worse intraretinal cysts/fluids compared with week 12  At week 20, 28, 32, 40, 44: decrease of ≥5 letters in comparison with week 12 due to nAMD activity.  **Maximal treatment period: eight weeks**  **Minimal treatment period: 12 weeks** | [21, 49] |

**Abbreviations:**  BCVA, best corrected visual acuity; CNV, subfoveal choroidal neovascularization ETDRS, early treatment diabetic retinopathy Study; FA, fluorescein angiography nAMD, neovascular age-related macular degeneration; OCT, Optical coherence tomography; T&E, treat and extend

**Table A3 Overview of the number of injections and number of patients in the different trials of the base case scenario**

| **Drug** | **Regime** | **Trials** | ***Year 1*** | ***Year 2*** | ***Year 1 + 2*** | ***Year 1+ 2 (corrected)*** | ***Number of patients (n)*** | ***Source*** |
| --- | --- | --- | --- | --- | --- | --- | --- | --- |
| ***Ranibizumab*** | **T&E** |  | **9.0** | **8.2** | - |  |  |  |
|  |  | *TREX* | 10.1 | 8.5 | 18.6 |  | 40 | [23, 40] |
|  |  | *LUCAS* | 8.0 | 8.0 | 16.0 |  | 172 | [13] |
|  |  | *TREND* | 8.7 | - | - |  | 294 | [24] |
|  |  | CANTREAT | 9.4 | 8.2 | 17.6 |  | 466 | [25, 41] |
| ***Bevacizumab*** | **T&E** | LUCAS | 9.0 | 9.2 | 18.2 |  | 167 | [13] |
| ***Aflibercept*** | **T&E** | ALTAIR^a^ | 7.2 | 3.5 | 10.4 | 10.7 | 108 | [22] |

Abbreviation: T&E, treat-and-extend.

^a^ The ALTAIR trial lasted 96 weeks. This time span was corrected to 24 months in order to align all results.
