# Supplementary material for: Cost-minimisation analysis of a treat-and-extend regimen with anti-VEGFs in patients with neovascular age-related macular degeneration
Source: Graefes Arch Clin Exp Ophthalmol. 2021 Oct 13;260(4):1083–95. doi: 10.1007/s00417-021-05359-x (PMC8511619; doi:10.1007/s00417-021-05359-x)
Supplement: Supplementary file 2 — (DOC 45.0 KB) [file 417_2021_5359_MOESM2_ESM.doc]

## **Appendix 2**

**Table A1 Description of the bevacizumab price calculations**

| One bevacizumab injection can be prepared out of the 4 ml flacons (25mg/ml) in the hospital pharmacy. An article on the costs of the preparation of bevacizumab eye injection from 2008, suggested that in optimal circumstances a hospital can prepare approximately 25 injections per flacon. Assuming that optimal circumstances are reached in the 12 years after this article has been published, this number was used in our model [27].  The costs of the preparation of bevacizumab injections described in this article were updated to the corresponding prices in November 2020 using the current Z-index, updated collective labour agreement of Dutch hospitals, and inflation rates [26, 43, 46]. The costs related to the quality assurance protocol described in the article were not included in the drug price calculations. It was assumed that nowadays most hospitals will not perform product specific controls. The costs for the preparation of bevacizumab include drug costs, personnel costs, overhead costs (e.g. the use of different rooms), and syringe costs. |
| --- |

**Table A2 Overview of the calculations of personnel the costs per bevacizumab preparation**

| **Personnel costs** | | | | | |
| --- | --- | --- | --- | --- | --- |
| **Functionary** | **Task** | **Costs per hour** | **Minutes** | **Costs per flacon** | **Source** |
| **Pharmacist assistant** | Working aseptically + labelling | €28.06 | 45 | €21.05 | [26, 43, 46] |
| **Production pharmacist** | Released protocol | €86.78 | 10 | €14.46 |
| **Laboratory pharmacist** | Released protocol | €86.78 | 10 | €14.46 |
| **Pharmacist quality assurance** | Formal issuing | €86.78 | 0 | - |
| **Microbiologist** | Sterility research | €30.31 | 0 | - |
| **Total personnel costs Total personnel including  overhead of 45%** | | | | **€49.47 €72.64** |  |

**Table A3 Overview of the calculations of personnel the costs per bevacizumab preparation**

|  | **Costs** | **Costs with inflation** | **Costs per injection** | **Source** |
| --- | --- | --- | --- | --- |
| **Costs per flacon** | €337.19 | €337.19 | €72.46 | [26, 43, 46] |
| **Costs per injections (average of 25 per flacon)** |  |  | €13.49 |
| **Syringe with needle** | €0.62 | €0.78 | €0.78 |
| **Personnel costs** |  |  | €2.90 |
| **Total: preparation costs + drug** | | | **€17.16** |  |
