# Supplementary material for: Cost-minimisation analysis of a treat-and-extend regimen with anti-VEGFs in patients with neovascular age-related macular degeneration
Source: Graefes Arch Clin Exp Ophthalmol. 2021 Oct 13;260(4):1083–95. doi: 10.1007/s00417-021-05359-x (PMC8511619; doi:10.1007/s00417-021-05359-x)
Supplement: Supplementary file 3 — (DOC 32.0 KB) [file 417_2021_5359_MOESM3_ESM.doc]

## **Appendix 3**

**Table A1 Overview of the number of injections and participants in the trial of scenario 2**

| **Drug** | **Regime** | **Trials** | ***Year 1*** | ***Year 2*** | ***Year 1 + 2 (after correction)*** | ***Number of patients (n)*** | ***Source*** |
| --- | --- | --- | --- | --- | --- | --- | --- |
| ***Brolucizumab (scenario 2)^a^*** | **QW8/QW12** | HAWK/HARRIER | 7.4 | 4.0 | 11.4 | 730 | [49, 70] |

^a^ The HAWK/HARRIER study lasted 96 weeks and only reported the total injection frequency over the total period. The injection frequency was corrected to a time period of 24 months and the number of injections were calculated by assuming that distribution was evenly after the loading dose.

**Table A2 Medication costs of brolucizumab**

|  | **Price** | **Source** |
| --- | --- | --- |
| **Brolucizumab ^a^** | € 825 | [70] |

**^a^** The Z-index prices was elevated with 9% because the Value Added Tax rate in the Netherlands (Dutch tax authorities 2020)
