# Supplementary material for: Cost-minimisation analysis of a treat-and-extend regimen with anti-VEGFs in patients with neovascular age-related macular degeneration
Source: Graefes Arch Clin Exp Ophthalmol. 2021 Oct 13;260(4):1083–95. doi: 10.1007/s00417-021-05359-x (PMC8511619; doi:10.1007/s00417-021-05359-x)
Supplement: Supplementary file 4 — (DOCX 28.0 KB) [file 417_2021_5359_MOESM4_ESM.docx]

**Fig A1 tornado diagram displaying the outcomes of the sensitivity analysis of aflibercept
Abbreviation:** OCT, Optical coherence tomography

**Fig A2 tornado diagram displaying the outcomes of the sensitivity analysis of bevacizumab
Abbreviation:** OCT, Optical coherence tomography

**Fig A3 tornado diagram displaying the outcomes of the sensitivity analysis of ranibizumab
Abbreviation:** OCT, Optical coherence tomography
